# 10 simple rules for teaching wet-lab experimentation to computational biology students, i.e., turning computer mice into lab rats

**DOI:** 10.1371/journal.pcbi.1007911

**Published:** 2020-06-04

**Authors:** Joseph C. Ayoob, Joshua D. Kangas

**Affiliations:** 1 Joint Carnegie Mellon–University of Pittsburgh PhD Program in Computational Biology, Pittsburgh, Pennsylvania, United States of America; 2 Department of Computational and Systems Biology, University of Pittsburgh School of Medicine, Pittsburgh, Pennsylvania, United States of America; 3 Computational Biology Department, Carnegie Mellon University, Pittsburgh, Pennsylvania, United States of America; Dassault Systemes BIOVIA, UNITED STATES

Graduate students in computational biology have strong quantitative backgrounds but are often quite limited in their understanding of the theory, approach, and practice of biological experimentation. A strong grasp of the provenance of relevant biological data is essential for computational biologists to effectively critique and incorporate data into their research efforts or to generate the data themselves, the latter of which is becoming more and more prevalent [1,2]. To give students this knowledge, insight, and experience, the joint Carnegie Mellon–University of Pittsburgh PhD program in Computational Biology (CPCB) has developed the Laboratory Methods for Computational Biologists (LMCB) course as a core course in the CPCB curriculum to provide a hands-on, research-oriented laboratory experience in four major areas: genomics, microscopy and bioimaging, high content screening, and X-ray crystallography. For each area, we have designed course modules covering general topics such as experimental design, limitations of common methods, cutting-edge and high throughput techniques, potential sources of error in data, and the preservation, analysis, and presentation of data. To provide the students with a more immersive research experience, we have designed the modules to cover one common research topic giving the students experience in using results from previous modules to inform the design of experiments for the next. The LMCB course provides foundational and experiential wet-lab training for the benefit of nascent computational scientists. Here, we provide some of the guiding principles and approaches that we’ve used to establish, evolve, and shape our course. While we use our course as the framework for the presented rules, they are broadly applicable and adaptable to similar graduate and undergraduate programs. Therefore, this article will appeal to anyone interested in approaches for providing hands-on training in experimental techniques to computational trainees, particularly to faculty and program directors of computational biology and related graduate or undergraduate programs that want to provide this foundational course-based research training to their students.

## Rule 1: Know why you are doing it (and make sure your students know too)

There are several motivators for offering our LMCB course and why we would encourage other computational programs to adopt similar course-based research experiences. Primarily, our educational goals align with the view that it is essential for our computationally focused students to understand and appreciate the nuances of the data they are using and how they were generated. This includes making them aware of the limitations and caveats of particular experimental approaches, importance of reproducibility, and overall quality and confidence level of the data. Moreover, we strive to enable our students to be able to better communicate with experimentalist collaborators by having them think like an experimentalist. Through these experiences, they are also better equipped to perform their own research one day and have multiple opportunities to network with the various experimentalists who are involved in teaching the course.

## Rule 2: Hire or identify an experimentalist to direct and teach the course

When computational research was first coming to prominence, many departments seemed to have one lonely computational person, who many may have seen as the square peg in the round hole. With the emergence of many computational biology departments, we would encourage these departments to now flip the script, if they haven’t already, and hire an experimentalist to bring that same diverse perspective to a more homogeneous environment and provide wet-lab training to the computational students therein. The authors of this article were both hired to primarily fulfill these roles in their respective departments. While we were hired for this specific task, we also serve in other teaching, research, administrative, mentoring, and outreach roles that align with the goals of our departments. For instance, we both serve as assistant directors for our master’s degree programs, direct summer programs for undergraduates and high school students, collaborate with computational colleagues, and have implemented a professional development course to hone the other essential skills that our students need to prepare themselves for success in the job market.

## Rule 3: Find space and collaborators

If your institution has dedicated laboratory space for teaching, this is a good place to start; however, these spaces may not have the proper equipment for all of the desired experiments, so a more distributed and/or nomadic approach to organizing this course can be advantageous. We have found that partnering with collaborators who have generous dispositions, the proper equipment, and lab space is a boon for the course. While we rely on our experience and skills for the bulk of the material for the course, there are other specialized areas that are beyond our expertise, and this is where the assistance from active researchers in these other fields is highly valuable for the student experience. For instance, we offer an X-ray crystallography module, in which we partner with Dr. William Furey, who teaches this portion of the class for our students and provides access to space and specialized equipment that we are able to use for X-ray crystallography experiments. Other faculty have also been willing to lend us some bench space and equipment for specific modules. By actively collaborating during this course, we have found that we can effectively broaden the scope outside the bounds set by instructor expertise and space and equipment availability. Strategic partnerships with neighboring departments, and potentially offering a course jointly with them, could also help bring in the necessary expertise to support and drive the course.

## Rule 4: Do real science

When our lab course was first started, there were many cookie cutter “experiments” and demonstrations that made up the curriculum. Because of this, student engagement was not high. It was rare to really see a student excited about, for example, using a kit to purify a protein for the sake of purifying a protein. Over the past several years, we have made a concerted switch to offering authentic research experiences for our students, and this made a huge difference in our course. We first moved to research-like experiences in which students would design and perform experiments to address particular questions within a framework that could be related to real research but not necessarily generate publishable data. This switch had a huge effect on the engagement of our students, so much so that we had to start kicking students out of the lab an hour past the end of class because they wanted to keep collecting data! Inspired by this success and the growing use of course-based undergraduate research experiences (CUREs) that focus on authentic, data-generating experiments in lab courses, we then moved to a central research question for the course—identifying novel approaches to mitigate the effects of snake venom toxicity [3,4]. This particular project was chosen because it is amenable to inquiry using the methods we have available, a paradigm that is rather simple to implement, and an exciting, yet neglected, topic in which our students’ work could have an impact [5]. We have organized the course for three of the four main modules to directly address this question, and expect to publish our results with our students as co-authors, which appears to have also helped with their level of engagement.

## Rule 5: Make it a real lab environment

We have incorporated many of the things that one would do in running a lab into our lab class. We hold journal clubs that have the students teach each other the important scientific concepts and experimental approaches that they will use in the lab. One of the assignments that wraps up the first two modules is a lab meeting, in which students present their results, which are critiqued together by the students and instructors. We also design the assignments to provide students with opportunities to practice their professional skills, such as writing, analyzing and presenting data, and talking about their research and its importance. We’ve even gone so far as to think about including the traditional, yet usually awkward, research group outings as a part of the course but can’t quite justify having “apple picking” or “laser tag” on our syllabus, yet.

## Rule 6: Let them drive

Students who have greater ownership of their projects and education are more invested and get more out of the experience [6–8]. We encourage a 2-fold approach to doing this. Incorporating active learning techniques into the lab class is a great way of engaging students in their own learning. For instance, to cover the vast number of approaches in genomics, cell biology, and bioimaging, we utilize jigsaw journal clubs [9] that have students identify review materials that cover a particular topic or approach, teach others about this topic in small groups, and then work together to answer research questions that draw on this new mutually-taught knowledge.

For the laboratory projects, we teach our students the basics of experimental design, provide them with a framework of a big picture research topic, and guide them through identifying what particular experiment(s) they want to perform and how to prepare for them given the reagents, equipment, and time available. While failure is often the norm initially for these experiments, these setbacks offer valuable lessons for students on their way to a more advanced understanding of laboratory experimentation.

## Rule 7: Be flexible

Doing experiments on a rigid time schedule (e.g., Tuesdays and Thursdays for two and a half hours) can be quite limiting and does not quite align well with an authentic research experience. This is especially true for multiday experiments and if there will be long delays in obtaining data, such as if you are sending out samples for sequencing. Being flexible in scheduling can provide a much richer research experience to the students. For instance, the LMCB course was once held entirely in the spring, two days a week for two and a half hours, going directly from one module to another. While we used this format, which is more amenable to an undergraduate course schedule, successfully for several years, when we decided to have most of our modules focus on one research question, we needed another model to allow time for data generation, reagent procurement, and planning, without filling the class with busy work. To address this, we spaced the research modules over both the fall and spring semesters with gaps between them and increased the frequency of the class meetings to three days a week during the modules to facilitate both the execution of multiday experiments and the repetition and mastery of techniques; we considered meeting every day, but that frequency was not needed for our experiments. With these changes, we have maximized educational utility while still maintaining a full semester of content.

## Rule 8: Prepare a budget

While it does take money to run any lab class, the investment can be worth it especially if the students are contributing to a genuine research project that is producing publication-quality data. There are also alternatives to squeezing a program budget to get funds. Educational discounts for reagents and equipment are helpful for defraying costs. Using external services for complex steps, such as next generation sequencing, can be relatively inexpensive as we are in the age of the sub-$1,000 genome. Buying your colleagues coffee and/or lunch could be a good trade for the use of some advanced equipment and piggybacking on some sequencing runs. The initial training grant that funded our graduate program included funds to purchase much of the equipment and consumables that we use in the course. Existing educational and infrastructure grant mechanisms are also possibilities. The budget should also incorporate some flexibility for discretionary spending for experimental reagents selected by students. While giving the students control over their experiments gives them more ownership of their projects, having them see the associated costs of reagents that they “buy” gives them an even greater level of stewardship for their class research.

## Rule 9: Always innovate

In the past 10 years of running the course, we have made major changes to the class by adding and removing modules as well as making extensive changes within modules: most of the modules have undergone major overhauls of approach and research questions at least twice. For instance, our genomics module went from doing some simple molecular biology experiments on a small scale to analyzing the microbiotas of cheeses and other fermented foods using the university’s Ion Torrent sequencing platform to our current experiments of performing RNA-seq analysis on cells treated with snake venom to detect RNA expression changes. These innovations have helped keep the class fresh for both the students and instructors.

## Rule 10: Keep it fun

Science is fun, so a class in which students get to do actual science should be fun too! We have found that students and instructors enjoy the course much more when the environment is loose. For instance, to teach students the basics of pipetting, we created a “Pipettapalooza” pipetting extravaganza during which students get to practice these essential skills using the tools and consumables they will use in the lab by playing games of Connect 4 and Battleship, honing their volume-visualization skills by attempting to replicate volumes of unknown size, and creating masterpieces or art using a pipette pointillism technique. Another engaging activity that we have used to teach experimental design is to have the class design an experiment to test whether carrots help improve one’s vision. This seemingly simple and silly example has provided some great discussion on what one needs to establish a carefully controlled experiment. Another tone-setting tool that we use is to set the expectation that their effective exploration of a question of their choosing is more important than getting the “right” answer. Dispelling students of the notion that they need to confirm their hypotheses in order to get a good grade helps relieve a lot of pressure off of them, lets them focus on doing things correctly, and opens their eyes to how to learn from failure. Lastly, we’re sure it wouldn’t hurt to take the class to go apple picking or play laser tag to make the experience all the more real.

## Conclusion

In our era of interdisciplinary research, nascent computational scientists must be versed in a diversity of areas that span biological, computational, mathematical, and physical sciences. The practical application of these disciplines, such as in an advanced laboratory class, provides real world experience that cannot be captured in a classroom and will directly aid our students to better tackle the emerging biological questions that lie ahead of them. To our knowledge, the LMCB course is the only such course that provides immersive training in genuine wet-lab research to computational biology students, and we would encourage similar programs to adopt this general model, align it with their strengths, and adapt it to the needs of their students. Implementing a laboratory class for computational scientists takes an important step beyond merely presenting the theory of experimentation and helps us live up to our duty of preparing the next generation of truly interdisciplinary scientists poised for success the fast-growing realm of computational biology.

## References

[1] Yanai I, Chmielnicki E. Computational biologists: moving to the driver's seat. Genome Biol. 2017; 18, 223. 29169371 10.1186/s13059-017-1357-1 PMC5701351

[2] Wright ES. Getting my feet wet. Science. 2017; 356 (6333), 106. 28386014 10.1126/science.356.6333.106

[3] Auchincloss LC, Laursen SL, Branchaw JL, Eagan K, Graham M, Hanauer DI et al Assessment of course-based undergraduate research experiences: a meeting report. 2017; 13, 29. 10.1187/cbe.14-01-0004 PMC3940459 24591501

[4] Science Education Resource Center at Carleton College. CUREnet. 2019. [cited 2019 Oct 28]. https://serc.carleton.edu/curenet/index.html.

[5] World Health Organization. Snakebite envenoming. [cited 2020 Feb 6]. https://www.who.int/health-topics/snakebite#tab=tab_1.

[6] Bonwell C and Eison JA. Active learning: creating excitement in the classroom. ASHE-ERIC Higher Education Report No. 1. Washington DC: The George Washington University, School of Education and Human Development; 1991.

[7] Prince M. Does active learning work? A review of the research. Journal of Engineering Education. 2013; 93, 223.

[8] Freeman S, Eddy SL, McDonough M, Smith MK, Okoroafor N, Jordt H et al Active learning increases student performance in science, engineering, and mathematics. Proceedings of the National Academy of Sciences. 2014; 111, 8410–8415. 10.1073/pnas.1319030111 PMC4060654 24821756

[9] Choe SWT, Drennan PM. Analyzing scientific literature using a jigsaw group activity: piecing together student discussions on environmental research. Journal of College Science Teaching. 2001; 30, 328.

